# Are Cisgender Women and Transgender and Nonbinary People Drinking More During the COVID-19 Pandemic? It Depends

**DOI:** 10.35946/arcr.v43.1.05

**Published:** 2023-12-28

**Authors:** Cindy B. Veldhuis, Noah T. Kreski, John Usseglio, Katherine M. Keyes

**Affiliations:** 1 Department of Medical Social Sciences, Feinberg School of Medicine, Northwestern University, Chicago, Illinois; 2 Institute for Sexual and Gender Minority Health and Wellbeing, Northwestern University, Chicago, Illinois; 3 Department of Epidemiology, Mailman School of Public Health, Columbia University, New York, New York; 4 Augustus C. Long Health Sciences Library, Columbia Irving Medical Center, Columbia University, New York, New York

**Keywords:** alcohol, cisgender women, transgender persons and nonbinary populations, sexual and gender minorities, college students, COVID-19, pandemic, culturally responsive treatment

## Abstract

**PURPOSE:**

This narrative review of research conducted during the first 2 years of the COVID-19 pandemic examines whether alcohol use among cisgender women and transgender and nonbinary people increased during the pandemic. The overarching goal of the review is to inform intervention and prevention efforts to halt the narrowing of gender-related differences in alcohol use.

**SEARCH METHODS:**

Eight databases (PubMed, APA PsycInfo, CINAHL, Embase, Scopus, Gender Studies Database, GenderWatch, and Web of Science) were searched for peer-reviewed literature, published between March 2020 and July 2022, that reported gender differences or findings specific to women, transgender or nonbinary people, and alcohol use during the pandemic. The search focused on studies conducted in the United States and excluded qualitative research.

**SEARCH RESULTS:**

A total 4,132 records were identified, including 400 duplicates. Of the remaining 3,732 unique records for consideration in the review, 51 were ultimately included. Overall, most studies found increases in alcohol use as well as gender differences in alcohol use, with cisgender women experiencing the most serious consequences. The findings for transgender and nonbinary people were equivocal due to the dearth of research and because many studies aggregated across gender.

**DISCUSSION AND CONCLUSIONS:**

Alcohol use by cisgender women seems to have increased during the pandemic; however, sizable limitations need to be considered, particularly the low number of studies on alcohol use during the pandemic that analyzed gender differences. This is of concern as gender differences in alcohol use had been narrowing before the pandemic; and this review suggests the gap has narrowed even further. Cisgender women and transgender and nonbinary people have experienced sizable stressors during the pandemic; thus, understanding the health and health behavior impacts of these stressors is critical to preventing the worsening of problematic alcohol use.

Although historically cisgender women (i.e., women whose sex assigned at birth is consonant with their gender) in the United States have had lower levels of alcohol consumption than cisgender men, recent analyses of historical and cohort data suggest that overall gender differences are narrowing.^1^ This narrowing is largely due to substantial increases in cisgender women’s alcohol use, binge drinking (operationalized as four or more drinks in 1 day for cisgender women; five or more drinks in 1 day for cisgender men)^1,2^ and alcohol use disorder (AUD; meets criteria for past 12-month dependence or abuse as established in the fifth edition of the *Diagnostic and Statistical Manual of Mental Disorders [DSM-V])*.^3^ Cisgender women also report more barriers to treatment^4,5^ and lower treatment utilization than cisgender men.^6–9^ Given that cisgender women may experience more severe alcohol-related problems (e.g., problems in relationships or at work^10^) and health impacts than do cisgender men, even at lower levels of alcohol use,^11^ understanding whether the pandemic has led to an increase in alcohol use among cisgender women is critically important.

Rates and risks for problematic alcohol use vary by sexual identity,^12–14^ race/ethnicity,^15^ and other factors, including socioeconomic status and relationship status.^16^ These differences may be partially explained by differences in stress levels, including economic stressors and psychological distress^17^ and may have been further modified by the coronavirus disease 2019 (COVID-19) pandemic. Research on mental health during the pandemic suggests that cisgender women experienced elevated rates of stress, anxiety, and depression compared to pre-pandemic rates,^18–20^ at least in the early stages of the pandemic. In contrast, some research has suggested no gender differences in pandemic-related emotional distress.^21,22^

Stress is one of the strongest predictors of substance use, including alcohol use,^23^ and higher levels of stressors increase risks for problematic alcohol use, including AUD.^24,25^ The COVID-19 pandemic often has been described as a “perfect storm” of multiple sources of stress and has been linked to worsened mental health and health behaviors overall.^21,22,26–29^ There is evidence of increased problematic alcohol use during previous pandemics;^30^ however, the COVID-19 pandemic is unique among recent pandemics in the breadth and duration of its impacts and thus may have more substantial effects on health and well-being, including alcohol use. Cisgender women, compared to cisgender men, may be particularly affected by the pandemic due to higher levels of stressors.^31,32^ These stressors may be related to negotiating working from home^28^ while balancing remote schooling for children,^21,28^ higher likelihood of working in frontline and/or caregiver jobs,^28,33^ increased risks for intimate partner violence,^34–38^ delays in accessing needed health care,^39^ isolation,^40–42^ and potentially higher risks for unintended pregnancies.^31^ In a prospective study of families, cisgender women, compared to cisgender men, reported higher levels of stressors across four out of five domains. Specifically, cisgender women experienced higher levels of stressors in work/finances (31% increase), home disruptions (64%), social isolation (13%), and health care barriers (94%).^42^ The burden of pandemic-related stressors, combined with chronic and cumulative stressors disproportionately impacting cisgender women (e.g., sexism and/or violence across the life span^43^), may result in allostatic overload, which heightens health risks.^44^ When faced with higher levels of stressors during the pandemic, cisgender women may be at higher risk than cisgender men for alcohol consumption because cisgender women are more likely than cisgender men to use alcohol to cope with negative emotions.^24,45^ Using alcohol to cope may have potentially disproportionate impacts on those experiencing the highest levels of stressors (e.g., frontline workers, parents).^42^

Transgender and nonbinary (TNB, i.e., people whose gender differs from their sex assigned at birth) individuals experience significant health disparities, and their health is negatively affected by high levels of stigma, discrimination, and violence, as well as low levels of support.^46–51^ The COVID-19 pandemic may have been particularly stressful for TNB people compared to cisgender people due to elevated socioeconomic impacts such as job loss,^52^ food^52^ and housing insecurity,^53,54^ as well as reductions in social and community support.^55–57^ TNB people also have experienced disruptions to medical care (including gender-affirming services), which heightens stress.^53,56^ Coping is a key motivation for alcohol use among TNB populations,^51,58,59^ which might suggest increased use of alcohol to cope during a stressful event such as a global pandemic. Yet, research findings on rates of alcohol use among TNB populations are more mixed compared to cisgender people.^60–64^ Problematic alcohol use is associated with increased risks for secondary harms that disproportionately affect TNB individuals, such as suicidal ideation, intimate partner violence, sexual violence, and the exacerbation of mental and physical health problems,^62,65,66^ highlighting the importance of a deeper understanding of alcohol use among TNB individuals. Additionally, TNB people experience barriers to treatment,^67^ including a lack of culturally responsive care options^68–73^ and discrimination by providers.^68^ Of note, the umbrella term “TNB” encompasses a diverse range of identities and experiences, but existing research often does not disentangle this diversity, instead aggregating across groups who fall outside of cis-normative gendered expectations and who then are compared with cisgender peers.

Understanding alcohol use among cisgender women and TNB people during the pandemic is particularly important due to risks for severe health outcomes. Not only are COVID-19 patients with AUD more likely to be hospitalized and to have higher all-cause mortality,^74^ but alcohol-related mortality spiked with the onset of the COVID-19 pandemic.^75,76^ Problematic alcohol use also is a major risk factor for COVID-19 infections and mortality.^77^ Although the connections between COVID-19 and alcohol use have widespread effects, specific alcohol-related health impacts of the pandemic have been particularly harmful for cisgender women, as indicated by a 125% increase in alcohol-associated hepatitis^78^ and a stark increase in the proportion of patients screening positive for substance use (including alcohol use) in emergency departments.^79^ To our knowledge, similar research has not been done among TNB populations.

This review aims to understand the unique experiences of cisgender women and TNB people, as well as among understudied groups of cisgender women such as women of color, sexual minority women (SMW, e.g., lesbian, bisexual, queer women), and older women to describe subgroup impacts of the COVID-19 pandemic on alcohol use. A recent scoping review of substance use during the pandemic noted the importance of examining substance use (including alcohol) during the pandemic among cisgender women and TNB populations.^80^ Thus, this review aims to evaluate the extant literature testing whether cisgender women drank at similar or higher levels than cisgender men during the pandemic. The review further explores alcohol use among TNB populations during the pandemic, with a focus on gender differences in rates of alcohol use (e.g., binge drinking, alcohol dependence, quantity/frequency of drinking) in research conducted during the pandemic (since March 2020) in the United States.

## Methods

### Search Methods Employed

This narrative review of alcohol use during the pandemic was conducted to document whether alcohol use had increased among women—a population already experiencing inclines in alcohol use before the pandemic—and among TNB people in order to inform needed prevention and interventions, as well as to inform policy. The review process included seven steps:^81–83^ (1) refining the topic and identifying the research question; (2) developing a protocol; (3) identifying relevant studies; (4) screening and selecting studies; (5) extracting the data; (6) critically appraising and synthesizing the data; and (7) reporting the results.

One author, a Health Sciences Library Informationist conducted the literature searches on July 15, 2022, in eight databases: PubMed (pubmed.gov); APA PsycInfo (EBSCO); CINAHL [Cumulative Index to Nursing and Allied Health Literature] (EBSCO); Embase (embase.com); Scopus (scopus.com); Gender Studies Database (EBSCO); GenderWatch (ProQuest); and Web of Science (webofscience.com). Because the review addresses two separate questions, two search strategies were used. The first strategy comprised a combination of search strings related to alcohol use, COVID-19, and women. The second strategy combined search strings for alcohol use, COVID-19, SMW, and TNB populations. No filters were applied to the search results.

All records found via the database searches were exported to an EndNote library (version X9). Duplicates were identified and removed in EndNote, and the remaining library was imported into the Covidence review software to facilitate identifying relevant articles for the narrative review. Articles were eligible for inclusion in this review if they met the following criteria hierarchically: (1) were published in peer-reviewed journals between March 2020 and July 2022; (2) were written in English; (3) used human participants in the United States (to reduce variability in responses to the pandemic); (4) included measurement of alcohol use (broadly defined); (5) collected data during the COVID-19 pandemic; and (6) included analyses of gender differences in rates of alcohol use or focused solely on cisgender women or TNB people and alcohol use during the pandemic. Articles were excluded if they were review papers or qualitative studies, if they did not conduct any gender differences analyses (unless the study focused on women or TNB samples only), and if alcohol was not an outcome.

### Data Extraction

**Figure 1:**
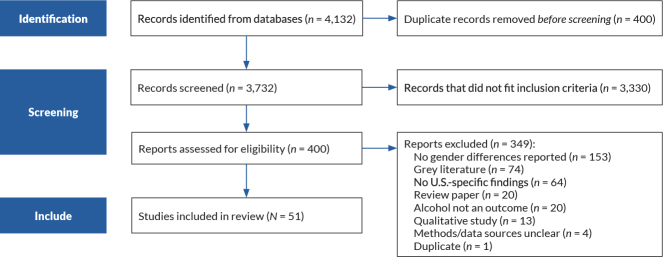
PRISMA flow diagram of search strategy used during the narrative review of women’s alcohol use during the pandemic. *Note:* PRISMA, Preferred Reporting Items for Systematic Reviews and Meta-Analyses.

After conducting a title and abstract review of all articles, the authors reviewed the full text of the remaining papers to determine final inclusion. Differences were discussed amongst three authors until agreement was reached. The full texts of the 400 articles were assessed for relevance to the review’s aims. When an article was excluded during the full review, authors documented the reason for its exclusion. (See Figure 1 for the search strategies for both questions combined.) Three authors critically reviewed and synthesized data from the 51 included articles.

## Results

### Results of the Literature Search

The literature search identified a total of 4,132 records. There were 400 duplicates, leaving 3,732 unique records for consideration in the review; of these, 51 articles ultimately were included.

### Results of the Reviewed Studies

Appendices 1 and 2 (located after the references) list the 51 reviewed articles and include all data from the abstraction protocol. Consistent with the goals of a narrative review, potential methodological limitations of the research are highlighted to help the reader better evaluate the validity and generalizability of the findings. The results are broken into four sections: (1) prevalence; (2) specific populations and demographic differences (age, race/ethnicity) or life experiences (pregnancy, intimate relationships, frontline work); (3) linkages between alcohol and mental health, stress, or coping; and (4) TNB individuals and SMW.

Table 1 includes descriptive data of the studies reviewed. Of those, 24% included nationally representative samples, 36% included pre-pandemic data (as opposed to retrospective reporting or only having within-pandemic data), 51% had data collection that ended early in the pandemic (March–May 2020), and 16% had data collection that ended in 2021. Slightly more than one-quarter (26%) used the Alcohol Use Disorders Identification Test (AUDIT) or AUDIT-Consumption (AUDIT-C), with several studies using just one or two items from the AUDIT. In addition, 6% used another validated measure, and 29% examined quantity and frequency only. Of those studies that looked at gender differences (as opposed to having a sample of cisgender women only), 73% found gender differences in alcohol use.

Of the 51 studies that met inclusion criteria, 20 studies tested for trends over time in alcohol use, including the pandemic period. Table 2 summarizes the results of those 20 studies, including the number of studies that found increases, decreases, or no change in alcohol use. Overall, 12 of the 20 studies documented increases in alcohol use during the pandemic period. More studies documented increases among cisgender women than among cisgender men (8 and 6 out of 13, respectively), and the only study with sufficient data to test for trends among TNB individuals found increases in alcohol consumption.

The following sections present the results in more detail, organized by prevalence data; specific subpopulations; stress, coping, and mental health; and alcohol use among SMW and TNB people. Not all studies had mutually exclusive samples; thus, studies may be mentioned in more than one section.

### Prevalence

Eighteen studies were primarily aimed at describing prevalence of alcohol use among adults during the pandemic and included analyses of gender differences. These studies were divided into two groups: cross-sectional studies (including repeated cross-sectional studies) of adults and longitudinal/prospective studies of adults.

#### Cross-sectional general population adult studies

Nine cross-sectional studies,^79,84–91^ all conducted during the early pandemic, met inclusion criteria. All used convenience samples, with two samples recruited from social media. In three studies that asked participants to compare retrospectively their pre-pandemic AUD symptoms to current symptoms,^85–87^ all found increased reports of AUD symptoms among cisgender women during the early pandemic compared with retrospective reports of pre-pandemic symptoms. In one study, cisgender men also reported increases;^85^ in another, they did not;^86^ and in the third study cisgender women reported increased drinking more often than did cisgender men.^87^ A fourth study found no gender differences in self-defined “drinking behaviors” during the early pandemic.^88^ Across these studies, the cross-sectional design—including retrospective reporting of pre-pandemic drinking behaviors and AUD symptoms as well as use of convenience samples based on volunteers from social media—limit the conclusions that can be drawn from these studies.

Three general population adult studies used repeated cross-sectional assessments (with different samples at each time point) before and during the pandemic to compare rates across time.^79,84,89^ Using nationally representative samples, Kerr et al.^89^ documented that daily drinking and alcohol volume were higher among cisgender women interviewed during the pandemic through 2021 compared to those interviewed pre-pandemic. AUD prevalence across the continuum from mild to severe was also higher during the pandemic. Sensitivity analyses indicated that results were robust to the timing of interviews and thus unlikely to be affected by pandemic-related social distancing policies. Electronic health record data of more than 100,000 patients visiting emergency departments showed higher alcohol admissions and evaluations for cisgender women during the pandemic compared with rates before the pandemic.^79^ In contrast, expenditure data, as an indirect measure of alcohol consumption, indicated lower household alcohol expenses during the pandemic, compared with pre-pandemic levels, for both cisgender men and cisgender women. However, expenditures may not correlate precisely with volume sales—for example, if purchases moved from on-premise to off-premise.^84^

Repeated cross-sections of data provide sufficient rigor for assessing changes in time trends, and all three studies included pre-pandemic time points, a notable strength. Given that two of the three studies found that increases in relatively serious alcohol-related harm (e.g., AUD, alcohol-related emergency department admissions) are concentrated among cisgender women, these data indicate an emerging concern.

#### Longitudinal general population adult studies

Nine longitudinal studies of adults in the general population met inclusion criteria.^92–100^ Three of these were based on a single data source, the Understanding America Study (UAS),^92,95,97^ a nationally representative panel study conducted monthly, with published data through mid-2020. All three studies from UAS demonstrated increases in alcohol consumption during the pandemic using repeated-measures longitudinal analyses, including increases in drinking days and near-daily drinking among cisgender women. However, these increases generally were less than those seen in cisgender men and remained below drinking levels among cisgender men.^92,95,97^ In a representative online sample of adults, among those who reported any alcohol use, cisgender men had higher levels of alcohol use (i.e., average number of drinks per day) than cisgender women at baseline (April–June 2019). However, alcohol use in cisgender men declined over time (last wave of data collection was in March 2021), whereas it stayed the same over time in cisgender women.^100^ In an additional nationally representative study with data from 2019 through the early months of the pandemic, days consuming alcohol and heavy drinking days (defined as five or more drinks within “a couple of hours” for cisgender men and four or more drinks for cisgender women) increased among cisgender women.^99^ Of note, however, no longitudinal studies of the general adult population included data beyond January 2021, and no studies published in 2022 met inclusion criteria for this review.

Given that surveys were completed by telephone both before and during the pandemic, it is unlikely that study methodology was substantially impacted by COVID-era research policies, although an impact on willingness to participate in research (either more or less willing) cannot be excluded and could be a limitation. However, taken together, the available research indicates that days consuming alcohol and heavy drinking days on average increased among cisgender women in the general population during the early and middle periods of the pandemic, but that for both variables, their consumption levels largely remained lower than, and did not change at the same rate as, those of cisgender men.

### Specific Populations and Demographic Differences

Several studies focused on unique subpopulations of cisgender women and alcohol use during the pandemic. The following sections discuss unique impacts on different age groups, different racial/ethnic populations, cisgender women in couple relationships, those who are pregnant or who are parents, and those who are frontline workers.

#### Adolescents, young adults, and older adults

Five cross-sectional studies met inclusion criteria; four^101–104^ were among young adult college undergraduates, and one was a nationally representative survey of high school students.^105^ No study had pre-pandemic data, and data collection spanned from early in the pandemic through early 2021. In the only nationally representative study of high school students meeting inclusion criteria,^105^ cisgender women students had higher rates of current alcohol consumption (defined as at least one drink in the past 30 days) than cisgender men students but did not report that they thought they drank more due to the pandemic. A cross-sectional survey of undergraduate college students conducted in fall 2020, with retrospectively reported pre-pandemic drinking, indicated increased consumption during the pandemic among all groups.^103^ Moreover, consumption and increases in consumption were greater among cisgender men compared with cisgender women and TNB individuals. Sexual minority groups generally reported higher levels of alcohol consumption and greater increases compared with pre-pandemic levels in both the high school and college samples; however, none of the studies examined interactions between sexual identity and gender. When coupled with the use of convenience samples, the cross-sectional designs and retrospective reporting limit inference from studies among college students.

Two studies included repeated cross-sectional samples of college students,^103,104^ one of which included pre-pandemic data collection.^103^ AUD prevalence was higher during the pandemic compared with pre-pandemic, with increases concentrated among cisgender women compared with cisgender men. For example, 49.7% of cisgender women met criteria for AUD during the pandemic, compared with 34.4% before the pandemic.

Seven studies^106–112^ included longitudinal data among young adults (two of the seven from the same data source^109,110^). All had pre-pandemic data points, a major strength of the evidence base. However, the span of pandemic data collection was limited to the early pandemic through late 2020. Two had nationally representative data (most used convenience samples).^108,112^ Most of these studies only reported data through spring 2020, which provides a limited assessment of pandemic-era changes in alcohol consumption, and findings regarding gender differences were mixed. Five of seven studies reported no gender differences in drinking as indicated by average past 3-month drinking quantity;^108^ self-assessment of changes in drinking during the pandemic; and binge drinking (i.e., five or more drinks in a row).^110,111^ A sixth study reported higher odds of drinking (any drinking on previous day) among cisgender men compared with cisgender women but noted no changes during the pandemic period.^112^

The remaining studies of college students and young adults generally found either faster declines in drinking among cisgender men,^106^ or faster increases,^109^ compared with cisgender women. A study comparing alcohol consumption during college spring semester across 3 years (2018, 2019, and 2020) found that whereas alcohol consumption (operationalized as number of drinking days and drinks per day) generally increased during spring semesters pre-pandemic, alcohol consumption either did not increase or declined in 2020 depending on the measure;^107^ no gender differences were found. The most robust studies (e.g., Jaffe et al. 2021^107^) indicate that college drinking largely declined in the early pandemic period, which is expected as students moved off campus, but there is little evidence for gender differences in these declines.

In sum, research among college students and young adults is mixed. Some studies found higher levels of alcohol use among cisgender men and some among cisgender women; however, overall, there were no increases in alcohol use among cisgender young women during the pandemic. Only one study identified for this review focused on older adults.^113^ In this study, which included a nationwide sample of older adults, cisgender women accounted for 59% of those who reported drinking more than usual during the pandemic.

#### Demographic differences by race/ethnicity

Only two studies focused on race/ethnicity and alcohol consumption during the pandemic.^114,115^ Among a sample of American Indian cisgender women followed prospectively through October 2021, approximately a quarter reported self-perceived increased consumption and half reported binge drinking (i.e., four or more “standard” drinks per day) during the pandemic.^114^ Among Black, indigenous, and other people of color (BIPOC) undergraduate students prospectively followed from before the pandemic through spring 2020, declines in drinking frequency were reported, but cisgender women, compared with cisgender men, were less likely to show declines.^115^ Overall, the sparse research is mixed on alcohol use among BIPOC cisgender women during the pandemic, suggesting that more research is needed.

#### Couple relationships and pregnancy

Three studies that met criteria for inclusion examined potential differences in alcohol use among cisgender women and their partners in heterosexual couple relationships and among cisgender pregnant women; one study also investigated how early parenthood might impact cisgender women’s alcohol use during the pandemic.^116–118^ The study of cisgender women and their men partners during the pandemic detected no gender differences in drinking levels; however, cisgender men reported more alcohol problems than did cisgender women. Cisgender women’s general stress and financial stress had no impacts on their partners’ drinking (drinks per week); however, cisgender men’s stress was associated with an increase in their partners’ drinking and a 22% increase in their own and their partners’ high-intensity drinking (defined as 10 or more drinks per day for men and eight or more drinks per day for women).^118^

There are mixed findings among pregnant cisgender women in reports of changes in alcohol use during the pandemic. Among a convenience sample of pregnant cisgender women, 11% reported perceived increases in their own and 28% in their partners’ alcohol use since the pandemic’s beginning. In contrast to these findings, none of the pregnant cisgender women in a study of centers for high-risk pregnancies reported self-perceived increases in alcohol use since the start of the pandemic.^116^ Notably, in the same study, 10% of postpartum cisgender women reported increased alcohol use.^116^

Together these findings suggest that in couple relationships during the pandemic, cisgender men’s stress levels and drinking may be associated with increased alcohol use and high intensity drinking among cisgender women. Findings among pregnant and postpartum women are mixed but suggest pregnancy and postpartum periods may heighten risk for some cisgender women. However, research was lacking on pregnant and postpartum TNB people during the pandemic, and further work should examine the impact of pregnancy more inclusively.

#### Frontline workers

Due to high levels of stress and risks for exposure to COVID-19 for health care and other frontline workers during the pandemic, research on health and health behaviors is important for understanding the broad impacts on this population. Yet, only two studies on frontline workers met inclusion criteria.^85,119^ Among health care workers in New Orleans, there were no significant gender differences in AUDIT-C scores. However, cisgender men’s rates of high-risk drinking (defined as a score of 4 or greater) stayed the same over time (45% at both time points), whereas cisgender women’s rates of high-risk drinking were higher during the pandemic compared to pre-pandemic (48% vs. 45%, respectively).^85^ In another study among health care workers at 25 hospitals, adjusted analyses found that cisgender women were no more likely than cisgender men to have symptoms consistent with probable AUD despite significantly higher likelihood of probable post-traumatic stress disorder (PTSD).^119^

### Coping, Stress, and Mental Health

The literature search yielded 10 studies that analyzed gender differences in alcohol use and also tested associations between stress or mental health and alcohol use during the pandemic.^94,100,119–126^ However, only five of these studies examined whether the associations between alcohol and stress or mental health differed by gender,^94,100,120,121,124^ three of which included pre-pandemic data.^100,120,124^ Two studies demonstrated mixed findings about drinking to cope early in the pandemic among cisgender women.^100,120^ One study found significant associations between COVID-related stressors and drinking to cope, with stronger associations for cisgender men than cisgender women.^120^ In the other study, stronger coping motives for drinking were associated with higher drinking levels at baseline for cisgender women, and loneliness and coping were related to changes in drinking levels over time.^100^

Analyses using data from a quasi-experimental study of a nationally representative sample determined that cisgender women interviewed during the pandemic (compared to cisgender women interviewed pre-pandemic) were nearly 1.5 times more likely to report that drinking helped them forget their worries.^124^ Among cisgender women, single women (compared to married women) were more likely, and Black women (compared to white women) were less likely to report drinking to forget their worries. Cisgender women with moderate to severe symptoms of depression (compared to no depressive symptoms; adjusted odds ratio: 2.45) and mild symptoms of anxiety (compared to no anxiety symptoms; adjusted odds ratio: 1.62) were significantly more likely to say that drinking helped them cope with their worries.^124^ There were no differences among cisgender men and no differences in comparisons between cisgender women and cisgender men. Depression and anxiety were associated with heightened risks for alcohol use^121^ and drinking to cope^124^ among cisgender women during the pandemic.

### TNB Individuals and SMW

#### TNB populations

Seven studies documented how the COVID-19 pandemic has impacted TNB people’s drinking.^101,115,127–131^ These studies included five cross-sectional and two prospective analyses, primarily began data collection in early pandemic, and all had trans-specific sample sizes of 200 or less. Within the literature that examined the drinking behaviors and trajectories of TNB people following the onset of COVID-19, the referent group to which TNB people were compared varied across studies. In some studies, the comparison was between TNB people and cisgender (or specifically cisgender and heterosexual) peers.^128,130,131^ In other studies, TNB people were aggregated and compared against cisgender women.^115,127,129^ One study included solely TNB people and evaluated their current behaviors against their retrospectively reported pre-pandemic behaviors.^101^

These comparisons provide differing information on TNB people’s drinking during the COVID-19 pandemic. Comparisons between TNB people and cisgender women, which were assessed at a variety of pandemic time points, typically found no significant differences between these groups in terms of alcohol use frequency (e.g., number of drinks consumed in a given day), alcohol use changes (e.g., self-reported drinking frequency before and during the pandemic), and likelihood of drinking to cope.^115,127,129^ For the literature comparing TNB populations to cisgender or cisgender/heterosexual peers more generally, TNB people and cisgender/heterosexual peers had comparable rates of increased drinking during the pandemic (TNB: 10.5%; cisgender/heterosexual: 13%) and were equally likely to exhibit problem drinking (based on PROMIS scores).^131^

Compared to cisgender men and SMW peers, TNB respondents reported a lower likelihood of problem drinking (using AUDIT),^130^ even though they reported higher psychological distress during the early pandemic.^128^ However, based on self-report, TNB respondents were more likely to report substantial increases in drinking during the pandemic. Notably, these results are drawn solely from college students.^130^

Other research on college students that drew from a more general sample addressed these substantial changes in drinking due to the pandemic, finding that mean number of drinks in the past 30 days among “non-cisgender” people, using the phrasing of that study, rose from 9.2 pre-pandemic (February 2020) to 16.8 during the pandemic (October 2020). However, these levels were lower than among either cisgender men or women peers.^101^ Extant research on TNB people’s drinking during the pandemic yielded conflicting results, with the most common result being null findings of differences between TNB people and cisgender peers across a number of drinking outcomes (though this varied based on the specific comparison being drawn). This small pool of research also lacked examinations of other TNB-specific factors that may influence drinking during the pandemic, such as transphobic experiences or sustained access to trans-related and trans-affirming health care as a preventive measure against psychological distress.

#### Sexual minority women

Four studies included findings specific to cisgender SMW.^127,128,132,133^ More SMW than any other group reported self-perceived increases in alcohol use since the start of the pandemic (39% vs. 33% of sexual minority men and 24.5% of cisgender heterosexual women).^133^ Two of the studies used the same sample but reported on different time points in recruitment (earlier in recruitment^132^ and after all participants had been recruited^127^). Among participants who were recruited earlier in the study/pandemic, most reported increased anxiety and depression since before the pandemic (more than 90%), but fewer reported increases in drinking (40% to 55% reported increases in drinking quantity, frequency, or both).^132^ Increases in anxiety and depression were associated with more alcohol consequences and motivation to drink to cope. In the analysis of the entire sample, participants indicated drinking on 26% of days as compared to using cannabis on 32% of days. On drinking days, participants consumed an average of almost three drinks per day and endorsed coping motives on 57% of drinking days.^127^ Overall, findings indicate higher incidence of increased alcohol use during the pandemic among sexual minority women compared to cisgender heterosexual women and sexual minority men; these increases were associated with higher risks for poor mental health. Notably, none of the studies reviewed included pre-pandemic data, and only one study was prospective.^127^ Two studies including sexual identity difference analyses (e.g., bisexual compared to lesbian cisgender women) within sexual minority women found few to no differences.^127,128^ Three studies included only young adults;^127,128,132^ only one study included participants from a wider age range (anyone older than age 18 was eligible).^133^

## Discussion

This review of the extant literature suggests that alcohol consumption, and especially reports of alcohol-related problems such as AUD symptoms, increased among adults in the United States during the pandemic. Although not all studies were entirely concordant, many increases in the most serious consequences of alcohol consumption seemed to be concentrated in cisgender women. That said, most studies, especially those representative of the U.S. population, indicate that alcohol consumption and alcohol-related harms remain higher among cisgender men. With respect to different subpopulations, data among young adults suggest that alcohol consumption in this age group declined in the early pandemic, with little evidence for gender differences in the decline. Too few studies have focused on cisgender BIPOC women, frontline workers, and older cisgender women to draw broader conclusions, suggesting a need for more research among these populations that have experienced stark disparities in the impacts of the pandemic.^33,42,134–138^

In the limited research that examined alcohol use among TNB populations, evidence suggests minimal differences in drinking frequency and other drinking outcomes (e.g., rates of increased drinking) between TNB and cisgender populations, at least when the comparison was between TNB people and either cisgender women or cisgender/heterosexual individuals.^115,127,129,131^ When compared with sexual minority college students, TNB college students had a lower likelihood of problem drinking (as determined using AUDIT) and a higher likelihood of self-reporting substantial changes in drinking during the pandemic.^130^ TNB college students exhibited increases in mean number of drinks in the past 30 days over the pandemic, but baseline levels were lower than in cisgender men and women peers.^101^ However, this body of research would benefit from clearer, more nuanced analyses that disentangle the rich diversity of TNB identities and stratify cisgender people by gender and sexual identity. Further research also is warranted on the specific experiences of TNB college students, as this population exhibited unique patterns. Additionally, research on pandemic drinking trajectories among TNB populations would benefit from a stronger emphasis on trans-specific experiences and stressors that may influence alcohol use; this research should be encouraged as an avenue of further inquiry.

Research among LGBTQ people during the pandemic broadly seems to suggest few to no differences compared with cisgender heterosexual populations.^104,139^ Notably, however, alcohol use seems to have increased since before the pandemic among sexual minority women,^133^ and these increases are associated with worsened mental health.^127,128^ This is an alarming finding given large pre-pandemic disparities in both alcohol use and mental health between sexual minority women and heterosexual women.^14,140–145^ More research is needed to understand the stressors and mechanisms underlying the higher rates of alcohol use among sexual minority women during the pandemic.

Efforts to combat elevated drinking must account for the complex reasons why people drink. Cisgender women were more likely to drink to help forget worries after (compared to before) the onset of the pandemic,^124^ and economic stressors—such as pay decreases, difficulty paying bills, or losing one’s job during the pandemic—have all been linked to increased drinking among cisgender women.^146^ Using alcohol as a coping mechanism impacted both TNB populations and cisgender women, as drinking to cope during the pandemic occurred at similar levels for both groups^127^ and was higher for TNB people and cisgender women than for cisgender heterosexual men.^147^ Cisgender women also experienced greater levels of unpaid labor (e.g., taking care of family members) during the pandemic, which may have increased stress levels.^31,148^ This may also be true for TNB people, who have faced distressing economic concerns and impacts^52,53,149^ as well as reduced access to health care, housing, and social/community support.^53–55,150^ Pandemic-related stressors may be particularly impactful for cisgender women’s drinking,^151^ but the potential impacts on TNB people’s drinking is less clear. Further research is needed to fully articulate any stressors and coping practices unique to TNB populations during the pandemic, such as potential shifts in proximal stress (e.g., anticipated stigma, concealment, or internalized transphobia), which has been linked to problematic alcohol use and drinking to cope.^58^

Whether the associations between mental health concerns and alcohol use were heightened during the pandemic is under-researched; however, rates of depression and anxiety have increased,^22,26,27,152^ which may put more people, particularly cisgender women, including SMW and TNB people, at higher risk of problematic alcohol use.

### Limitations of the Review

One key limitation of this review is the focus on alcohol; different forms of substance use can co-occur, potentially amplifying associated health risks.^80^ Research is limited on co-occurring substance use among cisgender women and TNB populations during the COVID-19 pandemic. Future research should address co-occurring substance use among cisgender women, sexual minority populations, and TNB populations to thoroughly examine its impact.

This review focuses solely on peer-reviewed publications, which may have led to a limitation of the research reviewed as only 16% of studies included time points in 2021 and none extended into 2022. Perhaps little research was conducted in 2021 that looked at the continued impacts of the pandemic on alcohol use; alternatively, findings may not yet be available in the peer-reviewed literature. Timing is important as different stages of the pandemic may have influenced population alcohol use heterogeneously; moreover, different geographic locations had discrete experiences of the pandemic. For example, the first case of COVID-19 in the United States was documented in January 2020 in Washington State, and cases were largely concentrated on the west coast until March 2020. Stay-at-home orders began in early to mid-March in some areas (e.g., Puerto Rico, California, New Jersey) whereas some states did not issue them until April (e.g., Iowa, South Carolina, Missouri).^153^ Many cities and states temporarily suspended bar and restaurant operations in the initial stages of the pandemic, which may have made alcohol less accessible; however, countervailing alcohol policies in many states that eased restrictions on take-out and home delivery of alcohol may have counteracted restrictions on on-premise consumption.^154,155^ Similarly, stressors associated with the initial stages of the pandemic could have contributed to higher rates of alcohol use compared with later stages of the pandemic. However, the extent to which stress eased as the pandemic continued remains understudied. Moreover, evidence suggests that boredom during the pandemic also may have been associated with increased alcohol use.^156,157^

Articles rarely mentioned when data collection occurred, much less with enough specificity to ensure it occurred during the pandemic, which made it difficult to screen out articles that collected data prior to 2020. To facilitate screening and identification of articles only looking at alcohol use during the pandemic, the authors made the decision to include “COVID” as part of the search strategy to capture relevant literature in the time available for the review and minimize the potential for not finding relevant studies. It would be beneficial to update this review in the future once more research has been published; however, this review gives a preliminary look at the available evidence.

This review excluded studies conducted outside of the United States, given the great variance in how different countries responded to the pandemic. Indeed, a recent systematic review suggests sizable variance in alcohol use during the pandemic depending on the country.^158^ This U.S.-centric review limited understanding of alcohol use by cisgender women and TNB people during the pandemic on a broader scale. Anecdotally, it was noted that many papers that examined gender differences or focused on cisgender women’s alcohol use were conducted outside of the United States. Future reviews should broaden the search to be inclusive of these important studies. Finally, the review excluded qualitative research, as the focus was on rates of alcohol use rather than on more nuanced findings related to reasons for alcohol use or experiences during the pandemic.

### Limitations of the Literature

Among the reviewed literature, the most robust designs were longitudinal, multi-cohort approaches and included pre-pandemic data (e.g., Jaffe et al.^107^). Pre-pandemic longitudinal data allow for assessment of pandemic-related deviations from existing patterns. For example, college students typically increase alcohol consumption during the spring semester; therefore, increases in alcohol use in spring 2020 during the pandemic period are not atypical and, in fact, might have been lower than expected.^107^ Another limitation is that most studies did not test for gender-by-time interactions; as a result, there are limited data on whether or not gender differences existed in changes over time. Examination of gender differences was further complicated by a frequent lack of clarity as to whether studies were reporting on sex or gender, or simply reporting on “women” without specifying how many of these women were cisgender or TNB. Generally, if studies did not mention TNB people in their study population, it is likely that TNB status was either not measured or considered, or that TNB people were actively excluded. Thus, in this review, studies that did not discuss gender outside of cisgender women and men, or that only used the terms “women” and “men,” were presumed to be not inclusive of TNB people.

Another limitation related to research design is measurement of alcohol use, changes in alcohol use, and other alcohol-related outcomes. Although many studies used validated measures of alcohol problems or commonly used measures of quantity and frequency, others relied on more subjective assessments. For example, 28% of the reviewed studies measured change in alcohol use by asking participants for their perceptions of change since the pandemic’s start, and 8% of studies asked participants to retrospectively report drinking levels pre-pandemic and current drinking. Retrospective subjective comparisons of alcohol use before and during the pandemic with unvalidated measures were perhaps necessary given the lack of pre-pandemic data collection in many studies but may have resulted in substantial measurement error. Further, definitions of alcohol use (e.g., problems, binge drinking) varied, making comparisons across studies challenging. Finally, given the heterogeneity of measures employed and domains of alcohol use examined, the current literature is limited in its ability to allow for any kinds of conclusions about differential rates of drinking versus alcohol problems.

Very few studies focused on BIPOC populations, which is particularly troubling given the sizable racial/ethnic disparities in COVID-19 infections and deaths^159^ and the compounding impacts of sociopolitical events, racism (including anti-Asian hate/attacks), xenophobia, and economic concerns on well-being.^160,161^ The review also found few studies that included comparisons between cisgender and TNB populations, and those that did lacked sample sizes to conduct subgroup comparisons among TNB people (e.g., transgender men versus transgender women), despite discrete risks.^64^ TNB populations are underrepresented in gender differences research; thus, more research on alcohol use among TNB people during the pandemic is needed to better understand rates of alcohol use and unique risk factors. Similarly, despite identified high risks among SMW, studies examining LGBTQ subgroups often had extremely small sample sizes for these groups, limiting the capacity for studies to identify significant differences. Few studies reported the intersections between gender and sexual identity (e.g., comparing bisexual men and bisexual women), thus limiting our understanding of gender differences.

No studies looked at gender differences in parenting and how that might be associated with potentially higher risk for alcohol use. Little research examined alcohol use among couples, despite ample research demonstrating partners’ impacts on each other’s drinking^162,163^ and clear linkages between intimate partner violence and alcohol,^164,165^ as well as the increased risks for intimate partner violence during the pandemic.^35,36,166^

One of the clearest limitations of the literature was the overall lack of research examining gender differences, which may be additionally related to the challenges of doing research during the height of the pandemic. The shift to working from home and the demands of social distancing made in-person research challenging, if not impossible, which had downstream implications for new research recruitment and data collection. Moreover, the pandemic had unequal impacts on the productivity of women and researchers from marginalized groups,^167–171^ which may have had disproportionate impacts on rates of research focused on cisgender women, BIPOC women, and TNB populations during the pandemic.

### Implications

The findings of this review point to a continued need for alcohol-reduction interventions. A discussion of the complexities of cisgender women’s and TNB people’s treatment utilization is beyond the scope of this review. However, there are unique pandemic-related considerations that may be worth attention. Although the extent to which pandemic-related increases in alcohol consumption will persist over the long term remains unknown, available research from disasters indicates that AUDs exacerbated by disaster exposures can persist over time for some individuals;^172^ thus, considering alcohol treatment and service capacity and pre-pandemic disparities is warranted. Interventions to reduce alcohol consumption and treat symptoms of AUDs have well-documented efficacy. However, before the pandemic, cisgender women^5^ and TNB individuals^62,67,173^ already had diminished rates of service utilization that may have been exacerbated in the pandemic setting. Digitally delivered services may increase access across populations,^174,175^ yet cisgender women, including SMW, and TNB people have more complex comorbidities that may require higher levels of care.^5,67,176,177^ For BIPOC women, SMW, and TNB people, treatment also needs to address minority stressors such as discrimination and stigma^51,173,178–183^ and needs to be intersectional to address the overlapping and compounding impacts of multiple sources of oppression and marginalization.^184–187^ Thus, an urgent research priority stemming from these findings is to evaluate accessibility and acceptance of service modalities.

There have been calls not to treat mental health concerns or problematic health behaviors as individual-level issues, particularly during a ubiquitous stressful and public health crisis such as a global pandemic.^188,189^ Instead, interventions should take a public health approach by modifying social and contextual factors to build resiliency.^160,190,191^ People have multiple motives for drinking, such as cravings^192^ or enhancing social situations.^112^ Yet, the unique impacts of pandemic-related stressors warrant enhancing access to resources, both emotional and economic, that may, in turn, help decrease stress- and coping-related motivations to drink. Efforts aimed at reducing distress and lowering risks for problematic alcohol use thus need to focus on ensuring consistent population-level access to resources such as social support, childcare and elder care, sick leave, affordable and accessible health care (including mental health care), affordable and permanent housing, education, living wages, and access to accurate health information. Whether these alone would be sufficient during a pandemic to reduce barriers to accessing help and uniquely support cisgender women and TNB people is unknown.

Further, alcohol policies to reduce access are effective in reducing harm.^193^ Alcohol policies generally became more permissive during the pandemic (e.g., “to-go” drinks, home delivery). Some of those pandemic-related changes are becoming permanent in some states.^194^ Revisiting alcohol regulation, including increasing price, as a public health approach could have considerable public health benefits.

### Summary of Conclusions

The gender gap in alcohol use is narrowing between cisgender men and women—and seems to have gotten even narrower during the pandemic. Additionally, cisgender women and TNB people are less likely to seek treatment, and there may be unique health risks related to COVID-19 and alcohol use at least for cisgender women. Thus, research, prevention, and intervention efforts are needed to address this public health issue. Halting this worrisome trend in alcohol use by cisgender women—across sexual identities—requires a public health approach that considers the unique needs and concerns of cisgender women. More research also is needed to understand alcohol use by TNB individuals during the pandemic and how to best build resilience and support for this underserved population. Ultimately, this paper is about both sex and gender, capturing the drinking-related experiences of cisgender women (for whom these align) and TNB populations (for whom they do not), as well as various subpopulations that may face unique risks (such as pregnant people). Thus, findings suggest that research on alcohol use and other mental health concerns needs to take both sex and gender (including gender-diverse individuals beyond just comparisons between cisgender men and women) into account to understand not only differences in rates and changes over time but also differences in predictors and outcomes.

## Figures and Tables

**Table 1:** Descriptives of Studies Included in Review

	*n*	%
**Data collection start**		
Early pandemic (March–May 2020)	26	51.0%
Late 2020	7	13.7%
Pre-pandemic	18	35.3%
**Data collection end**		
Early pandemic	26	51.0%
Late 2020	17	33.3%
Early 2021	7	13.7%
Late 2021	1	2.0%
**Study design**		
Prospective	20	39.2%
More than one cross-sectional time point	7	13.7%
Cross-sectional	24	47.1%
**Samples included**		
Cisgender women only	4	7.8%
Cisgender women and men	33	64.7%
Cisgender women, men, and TNB people	4	7.8%
Cisgender women and TNB people	10	19.6%
**Comparison groups**		
Cisgender men	36	70.6%
TNB individuals	1	2.0%
Cisgender men and TNB individuals	9	17.6%
No comparison group	5	9.8%
**Sample recruitment**		
Nationally representative	12	23.5%
Convenience	8	15.7%
Convenience: Online/social media	20	39.2%
Clinic sample	5	9.8%
Undergraduates (various recruitment methods)	5	9.8%
Other	1	2.0%
**Drinking measurement**		
AUDIT or AUDIT-C	13	25.5%
Daily drinking questionnaire	3	5.9%
Quantity and frequency	15	29.4%
Quantity	3	5.9%
Frequency	7	13.7%
Perceptions	5	9.8%
Other validated scale	3	5.9%
Other	2	3.9%
**How change was measured**		
Pre- and post/during pandemic data	10	19.6%
Retrospective recall of pre-pandemic AUDIT	1	2.0%
Retrospective report of current drinking in past vs drinking now	4	7.8%
Self-perceived changes in alcohol use	14	27.5%
Self-report of current drinking at more than one time point	12	23.5%
Did not measure changes in drinking	10	19.6%

*Note:* AUDIT, Alcohol Use Disorders Identification Test; AUDIT-C, AUDIT-Consumption; TNB, transgender or nonbinary

**Table 2:** Summary of Results for Changes in Drinking After Onset of COVID-19 Pandemic

	Number of Possible Studies	Proportion With Finding*	
		*n*	%
**Overall**			
Alcohol use or problems increased	20	12	60.0%
Alcohol use or problems decreased	20	5	25.0%
Alcohol use or problems did not change	20	3	15.0%
**Cisgender Women**			
Alcohol use or problems increased	13	8	61.5%
Alcohol use or problems decreased	13	2	15.4%
Alcohol use or problems did not change	13	3	23.1%
**Cisgender Men**			
Alcohol use or problems increased	13	6	46.2%
Alcohol use or problems decreased	13	3	23.1%
Alcohol use or problems did not change	13	4	30.8%
**Transgender and Nonbinary Individuals**			
Alcohol use or problems increased	1	1	100%
Alcohol use or problems decreased	1	0	0%
Alcohol use or problems did not change	1	0	0%

* Percentages within each group may not total 100% due to rounding. *Note:* COVID-19, coronavirus 2019.

**Appendix 1:** Description of Studies Included in This Review (*N* = 51): Sample Sizes, Recruitment Methods, Study Design, and Timing of Start and Stop of Data Collection*

#	First Author	Year	*N*	Sample Sizes of Subgroups	Sample	Recruitment	Study Design	Data Collection†	
								Start	End
Prevalence: Single and Repeated Cross-Sectional Studies of General Population Adults									
1	Chandran^79^	2021	107,930	57% cisgender women	EHR data; no age restrictions	Electronic health records	More than one cross-sectional time point	Pre-pandemic	Early pandemic
2	Acharya^84^	2022	18,808	54% cisgender women	Adults	Consumer data	More than one cross-sectional time point	Pre-pandemic	Late 2020
3	Beiter^85^	2022	102	48% cisgender women	Adult health care workers	Convenience	Cross-sectional	Early pandemic	Early pandemic
4	Boschuetz^86^	2020	417	84% cisgender women	Adults	Convenience: Online/social media	Cross-sectional	Early pandemic	Early pandemic
5	Capasso^87^	2021	5,850	53% cisgender women	Adult social media users in U.S.	Convenience: Online/social media	Cross-sectional	Early pandemic	Early pandemic
6	Grossman^88^	2020	832	84% cisgender women	Adults	Convenience: Online/social media	Cross-sectional	Early pandemic	Early pandemic
7	Kerr^89^	2022	1,819	52% cisgender women	Adults	Nationally representative	More than one cross-sectional time point	Pre-pandemic	Early 2021
8	Knell^90^	2020	1,809	67% cisgender women	Adults living in U.S.	Convenience: Online/social media	Cross-sectional	Early pandemic	Early pandemic
9	Walia^91^	2021	3,865	50% cisgender women	Health Information National Trends Survey	Nationally representative	Cross-sectional	Early pandemic	Late 2020
Prevalence: Longitudinal/Prospective Studies of General Population Adults									
1	Chartier^92^	2021	5,874	51% cisgender women	Adults	Nationally representative	Prospective	Early pandemic	Early pandemic
2	French^93^	2022	2,040	58% of sample at both time points were cisgender women	Adults living in U.S.	Convenience: Online/social media	Prospective	Early pandemic	Early pandemic
3	Lannoy^94^	2022	86	47% cisgender women	People who are HIV+, people with AUD, people with both, and controls with neither	Clinical sample recruited from longitudinal study	Prospective	Early pandemic	Early 2021
4	Leventhal^95^	2022	8,130	52% cisgender women	Adults	Nationally representative	Prospective	Early pandemic	Early 2021
5	Meanley^96^	2022	2,121	58% cisgender women	Participants from two prospective observational cohort studies	Pulled from MACS and WIHS cohorts	Prospective	Pre-pandemic	Late 2020
6	Nordeck^97^	2021	4,298	49% cisgender women	Adults	Nationally representative	Prospective	Early pandemic	Late 2020
7	Osaghae^98^	2021	267	72% cisgender women	Outpatient primary care clinic patients who had received a COVID-19 test	Clinic sample	Prospective	Early pandemic	Early pandemic
8	Pollard^99^	2020	1,540	57% cisgender women	Adults	Nationally representative	Prospective	Pre-pandemic	Early pandemic
9	Tucker^100^	2022	1,118	52% cisgender women	Participants from RAND ALP study had to report past-year alcohol use.	Nationally representative	Prospective	Pre-pandemic	Early 2021
Specific Populations and Demographic Differences: Adolescents, Young Adults, and Older Adults									
1	Coakley^101^	2021	777	62% women; 4% non-cisgender; 31% non-heterosexual	College students	Convenience sample of undergraduates	Cross-sectional	Late 2020	Late 2020
2	Hill^102^	2022	501	71% cisgender women; 0.6% nonbinary or transgender	College students living in U.S.	Undergraduate research pool	Cross-sectional	Late 2020	Early 2021
3	Kim^103^	2022	Pre-pandemic: 3,643; Pandemic: 4,970	Pre-pandemic survey: 70% cisgender women, 4% TNB Pandemic survey: 68% cisgender women, 2% TNB	College students	All first- and second-year undergraduates	More than one cross-sectional time point	Pre-pandemic	Early pandemic
4	Schwartz^104^	2022	526	74% cisgender women	College students	Convenience: Online/social media	More than one cross-sectional time point	Early pandemic	Late 2020
5	Brener^105^	2022	7,705	Not reported	Grades 9–12	Nationally representative	Cross-sectional	Early pandemic	Late 2021
6	Graupensperger^106^	2021	572	61% cisgender women	Young adults reporting at least one alcoholic beverage in past year	Convenience	Prospective	Pre-pandemic	Early pandemic
7	Jaffe^107^	2021	1,365	Not reported	College students	Undergraduate research pool	Prospective	Pre-pandemic	Early pandemic
8	Miech^108^	2021	582	51% adolescent girls	12th graders from Monitoring the Future survey	Nationally representative	Prospective	Pre-pandemic	Late 2020
9	Romm^109^	2022	1,084	51% cisgender women; 3% “other”	Young adults	Convenience: Online/social media	Prospective	Pre-pandemic	Late 2020
10	Romm^110^	2021	1,082	51% cisgender women; 3% “other”	Young adults	Convenience: Online/social media	Prospective	Pre-pandemic	Late 2020
11	Ryerson^111^	2021	302	2019 survey: 64% cisgender women 2020 survey: 68% cisgender women	College students	Undergraduates in health classes	Prospective	Pre-pandemic	Early pandemic
12	Stevenson^112^	2021	633	43% cisgender women	Young adults	Nationally representative	Prospective/daily diary	Pre-pandemic	Early pandemic
13	Eastman^113^	2021	6,938	54% cisgender women	U.S. adults age 55 and older	Nationally representative	Cross-sectional	Early pandemic	Early pandemic
Specific Populations and Demographic Differences: Race/Ethnicity									
1	Hanson^114^	2021	62	100% cisgender women	American Indian women	Sample from RCT	Prospective	Pre-pandemic	Early pandemic
2	Hicks^115^	2022	323	77% cisgender women; 4% TNB participants	Racial/ethnic minority undergraduate students	Convenience: Online/social media	Prospective	Pre-pandemic	Early pandemic
Specific Populations and Demographic Differences: Frontline Workers									
1	Beiter^85^	2022	102	48% cisgender women	Adult health care workers	Convenience	Cross-sectional	Early pandemic	Early pandemic
2	Hennein^119^	2021	1,092	72% cisgender women	Health care workers at teaching hospitals	Convenience	Cross-sectional	Early pandemic	Early pandemic
Specific Populations and Demographic Differences: Couple Relationships, Pregnancy, and Parenting									
1	Ahlers-Schmit^116^	2020	114	100% cisgender women	Convenience sample of pregnant women or mothers of infants	Convenience	Cross-sectional	Early pandemic	Early pandemic
2	McMillan^117^	2021	49	100% cisgender women	Women age 18 and older who were at least 12 weeks pregnant	Convenience: Online/social media	Cross-sectional	Late 2020	Late 2020
3	Rodriguez^118^	2021	118 couples	50% cisgender women	U.S. adults who consumed at least 12 alcoholic beverages in past year and live with partner	Convenience: Online/social media	Cross-sectional	Late 2020	Late 2020
Coping, Stress, and Mental Health									
1	Lannoy^94^	2022	86	47% cisgender women	People who are HIV+, people with AUD, people with both, and controls with neither	Clinical sample recruited from longitudinal study	Prospective	Early pandemic	Early 2021
2	Tucker^100^	2022	1,118	52% cisgender women	Participants from RAND ALP study who had to report past-year alcohol use	Nationally representative	Prospective	Pre-pandemic	Early 2021
3	Hennein^119^	2021	1,092	72% cisgender women	Health care workers at teaching hospitals	Convenience	Cross-sectional	Early pandemic	Early pandemic
4	Cummings^120^	2021	2019: 247;2020: 868	February 2019: 45% cisgender women; 0% transgender; 2% gender fluid March 2020: 52% cisgender women; 0.3% transgender; 0.6% gender fluid	Adults living in U.S.	Convenience: Online/social media	More than one cross-sectional time point	Pre-pandemic	Early pandemic
5	Devoto^121^	2022	499	100% cisgender women	Adult women living in U.S. who agree to share Facebook data	Panel	Cross-sectional	Late 2020	Late 2020
6	Graupensperger^122^	2021	1,181	60% cisgender women	College students	Convenience	Cross-sectional	Early pandemic	Early pandemic
7	Helminen^123^	2021	68	100% cisgender women	Community sample of trauma-exposed adult women	Convenience	Cross-sectional	Early pandemic	Late 2020
8	Martinez^124^	2022	Pre-pandemic: 1,291; Early pandemic: 812	61% cisgender women at baseline	Two cross-sectional NAS samples	Nationally representative	More than one cross-sectional time point	Pre-pandemic	Early pandemic
9	Nesoff^125^	2021	2,175	85% cisgender women; 4% TNB people	Adults living in U.S.	Convenience: Online/social media	Cross-sectional	Early pandemic	Early pandemic
10	Vogel^126^	2021	180	65% cisgender women	Recruited through Qualtrics	Convenience: Online/social media	Cross-sectional	Early pandemic	Late 2020
Transgender and Nonbinary Populations									
1	Coakley^101^	2021	777	62% women; 4% non-cisgender; 31% non-heterosexual	College students	Convenience sample of undergraduates	Cross-sectional	Late 2020	Late 2020
2	Hicks^115^	2022	323	77% cisgender women; 4% TNB participants	Racial/ethnic minority undergraduate students.	Convenience: Online/social media	Prospective	Pre-pandemic	Early pandemic
3	Dyar^127^	2022	429	73% cisgender women; 15% nonbinary; 5% genderqueer; 4% nonconforming; 3% “another identity”	Same criteria as Dyar 2021 study	Convenience: Online/social media	Prospective	Late 2020	Early 2021
4	Salerno^128^	2021	509	78% AFAB: 69% cisgender, 9% transgender, 1% nonbinary, 0.9% queer gender	Sexual and gender minority full-time college students	Convenience: Online/social media	Cross-sectional	Early pandemic	Late 2020
5	Sumetsky^129^	2022	247	59% cisgender women, 15% TNB	Adults in Allegheny County, PA	Convenience: Online/social media	Cross-sectional	Early pandemic	Late 2020
6	Zhang^130^	2022	366	47% cisgender women, 4% trans women, 8% trans men, 15% nonbinary, 2% genderqueer, 3% another gender	LGBTQ+ college students	Convenience: Online/social media	Cross-sectional	Early pandemic	Early pandemic
7	Akré^131^	2021	3,245	84.9% cisgender straight; 3.7% cisgender gay or lesbian; 7.0% cisgender bisexual; 3.8% cisgender men who have sex with men and women who have sex with women but do not identify as LGBT; 0.6% transgender	Adults in Atlanta, GA; Chicago, IL; New Orleans, LA; New York, NY; and Los Angeles, CA	Panel	Cross-sectional	Early Pandemic	Late 2020
Sexual Minority Women									
1	Dyar^127^	2022	429	73% cisgender women; 15% nonbinary; 5% genderqueer; 4% nonconforming; 3% “another identity”	Same criteria as Dyar 2021 study	Convenience: Online/social media	Prospective	Late 2020	Early 2021
2	Salerno^128^	2021	509	78% AFAB and 69% of sample was cisgender; 9% transgender; 1% nonbinary; 0.9% queer gender	Sexual and gender minority full-time college students	Convenience: Online/social media	Cross-sectional	Early pandemic	Late 2020
3	Dyar^132^	2021	212	74% cisgender women; 18% genderqueer or nonbinary; 9% another gender	Age 18–25; live in U.S.; lesbian, bisexual, pansexual, or queer; AFAB; reported four or more drinks at least twice and/or using cannabis in past month	Convenience: Online/social media	Prospective/EMA/daily diary study	Late 2020	Early 2021
4	Peterson^133^	2021	170	64% cisgender women	U.S. Adults	Convenience: Online/social media	Cross-sectional	Early pandemic	Early pandemic

Articles are listed in the order in which they appear in the manuscript. Some studies are listed in more than one section of the table.

† Time periods for start and stop of research studies: Pre-pandemic (Before March 2020); Early pandemic (March–May 2020); Late 2020 (June–December 2020); Early 2021 (January–May 2021); Late 2021 (June–December 2021).

*Note:* AFAB, assigned female at birth; AUD, alcohol use disorder; COVID-19, coronavirus disease 2019; EHR, electronic health record; EMA, ecological momentary assessment; HIV+, human immunodeficiency virus–positive; LGBT, lesbian, gay, bisexual, transgender; LGBTQ+, lesbian, gay, bisexual, transgender, and queer or questioning; MACS, Multicenter AIDS Cohort Study; NAS, National Alcohol Survey; RAND ALP, RAND American Life Panel; RCT, randomized controlled trial; TNB, transgender and nonbinary; WIHS, Women’s Interagency HIV Study.

**Appendix 2:** Description of Studies Included in This Review (*N* = 51): Measurement of Alcohol Use and Changes in Alcohol Use and Brief Findings*

#	First Author	How was alcohol use measured?	How were changes in alcohol use measured?	Gender Differences	
				Gender differences?	Findings
Prevalence: Single and Repeated Cross-Sectional Studies of General Population Adults					
1	Chandran^79^	SBIRT and intoxication admissions; AUDIT	Pre- and post/during pandemic data	Yes	Weekly SBIRT screens similar across gender in the pre-pandemic wave, then increased more for cisgender women than cisgender men.
2	Acharya^84^	Bi-weekly alcohol expenditures	Pre- and post/during pandemic data	No	Both cisgender men and women had decreased in spending on alcohol during pandemic, gender differences in spending during pandemic were not significant.
3	Beiter^85^	AUDIT	Retrospective recall of pre-pandemic AUDIT	Yes	Cisgender men higher AUDIT than cisgender women; all reported increases in AUDIT compared with pre-pandemic; no gender by time interaction assessed.
4	Boschuetz^86^	AUDIT-C, quantity and frequency, binge drinking	Retrospective report of current drinking in past vs. drinking now	Yes	Cisgender women reported more AUDIT-C symptoms after start of pandemic, cisgender men did not; no changes in alcohol frequency.
5	Capasso^87^	Self-perceptions of change in alcohol use	Self-perceived changes in alcohol use	Yes	Among those who reported increased drinking, 61% were cisgender women compared to 39% who were cisgender men (statistically significant). Younger participants more likely to report increased drinking, but no interactions examined between age and gender.
6	Grossman^88^	Days consumed, drinks consumed, binge drinking	N/A	No	No gender differences in number of days consumed alcohol, total drinks, or binge drinking.
7	Kerr^89^	Graduated frequency series. *DSM-V* AUD criteria	Pre- and post/during pandemic data	Yes	Daily drinking increased for both cisgender men and women, as did AUD mild and moderate/severe; moderate/severe AUD increased more for cisgender women than for men; volume, especially wine and spirit volume, increased more for cisgender women than men.
8	Knell^90^	Ever use and current quantity and frequency from BRFSS	Self-perceived changes in alcohol use	No	No gender differences in self-perceptions of changes in alcohol use since start of pandemic.
9	Walia^91^	Quantity	N/A	Yes	Significant gender differences, but no pairwise differences reported. Cisgender men had double the rates of reporting 13 or more drinks in a week than did cisgender women; other drinking levels did not differ.
Prevalence: Longitudinal/Prospective Studies of General Population Adults					
1	Chartier^92^	Alcohol use frequency	Self-report of current drinking at more than one time point	Yes	June 2020: cisgender women drank less than cisgender men; in change models, increased drinking during the month was no different between cisgender men and women, but cisgender women less likely to decrease drinking.
2	French^93^	“In the past three months, has alcohol consumption increased, stayed the same, or decreased?”	Self-perceived changes in alcohol use	Yes	Cisgender women significantly less likely than cisgender men to say that alcohol consumption had increased.
3	Lannoy^94^	AUDIT	Pre- and post/during pandemic data	No	No sex differences, stable AUDIT scores between assessments
4	Leventhal^95^	Frequency and intensity of drinking	Self-report of current drinking at more than one time point	Yes	Cisgender women comprised higher percentage of minimal and moderate/late decreasing trajectory group; lower percentage in moderate/early increasing, and near daily/early increasing
5	Meanley^96^	Reported frequency with which they consumed at least five (cisgender women) or six (cisgender men) alcoholic beverages in one sitting.	Pre- and post/during pandemic data	Yes	Cisgender men significantly more likely to be in the ‘any binge drinking’ trajectory group. Significant gender by time interaction; both cisgender men and women exhibited significant binge drinking decreases at time three compared to time one; decrease larger in cisgender men.
6	Nordeck^97^	Number of drinking days per week	Self-report of current drinking at more than one time point	Yes	Cisgender women had lower number of drinking days overall; both cisgender women and men increased drinking days; cisgender men increased more.
7	Osaghae^98^	AUDIT-C	Self-report of current drinking at more than one time point	Yes	36.1% of cisgender women and 32.9% of cisgender men reported hazardous drinking at baseline. Did not test gender by time interaction.
8	Pollard^99^	Days drank, number of drinks, heavy drinking days	Self-report of current drinking at more than one time point	Yes	Days consumed increased more for cisgender women; number of drinks increased more for cisgender men; heavy drinking days increased more for cisgender women; SIP scale not different
9	Tucker^100^	Quantity and frequency; Alcohol problems assessed with the Short Inventory of Problems^195^	Pre- and post/during pandemic data	Yes	Analyses were stratified by gender. Cisgender men’s alcohol use started out higher than cisgender women but declined whereas cisgender women’s stayed static. By time 3, drinking levels were about the same. Both cisgender men and cisgender women had increased alcohol problems over time. Coping and social reasons for drinking and loneliness had distinct associations with alcohol use, alcohol problems, and change over time and these varied by gender.
Specific Populations and Demographic Differences: Adolescents, Young Adults, Older Adults					
1	Coakley^101^	Quantity and frequency	Self-report of current drinking at more than one time point	Yes	Pre-pandemic (retrospectively reported), cisgender men drank more than cisgender women who drank more than TNB participants; during pandemic, consumption increased across groups, but remained cisgender men > cisgender women > TNB; cisgender men and TNB participants had greatest percent change during pandemic.
2	Hill^102^	AUDIT	N/A	Yes	Cisgender men had higher AUD symptoms than cisgender women. No pre-pandemic data and no time by gender interaction tested.
3	Kim^103^	AUDIT	N/A	Yes	Increases in AUD more concentrated among cisgender women
4	Schwartz^104^	“During the last two months, how often have you engaged in alcohol use?”	Retrospective report of current drinking in past vs drinking now	No	Gender differences tested but not significant. Alcohol use worsened between spring and fall 2020.
5	Brener^105^	Quantity and frequency, current binge drinking	Self-perceived changes in alcohol use	Yes	Cisgender women higher than cisgender men for current and binge drinking; no differences in perceived changes since pandemic. Sexual minority students reported higher current alcohol use, binge drinking, and drinking during the pandemic than did heterosexual students.
6	Graupensperger^106^	Quantity/frequency; Drinks per occasion	Self-report of current drinking at more than one time point	Yes	At baseline, cisgender women lower drinking than cisgender men; drinking declined at follow-up; declines were greater for cisgender men than cisgender women (significant interaction).
7	Jaffe^107^	Quantity and frequency	Self-report of current drinking at more than one time point	Yes	Cisgender men greater drinking days, greater drinks per day (both across years and within 2020); college students did not increase drinking in spring 2020 as was typical in previous years; no gender by time interaction reported.
8	Miech^108^	“Think back over the last 2 weeks. How many times have you had five or more drinks in a row?”	Pre- and post/during pandemic data	No	Study found that past 2-week binge declined from spring to summer 2020 overall; no overall gender differences; did not test time by gender interaction.
9	Romm^109^	Past 30-day quantity and frequency	Self-report of current drinking at more than one time point	Yes	Baseline drinking was lower for cisgender men than cisgender women; increases in alcohol use during pandemic greater for cisgender men than cisgender women
10	Romm^110^	“Compared to before COVID-19, are you doing more or less of the following: drinking alcohol?”	Self-perceived changes in alcohol use	No	41.3% of participants reported increased alcohol use; no gender difference in self-reported increased alcohol use
11	Ryerson^111^	Typical total weekly volume of alcohol consumption	Self-report of current drinking at more than one time point	No	No gender differences in alcohol consumption; 2020 cohort decreased alcohol consumption compared with 2019 cohort, especially those > 21; gender interaction with time was statistically significant, but direction not reported.
12	Stevenson^112^	Any drinking; drinking intensity on drinking days	Self-report of current drinking at more than one time point	Yes	Cisgender men more likely to report any drinking; no change in drinking during COVID; no gender interaction reported.
13	Eastman^113^	“Over the past week, have any of your usual daily activities or behaviors changed?”	Self-perceived changes in alcohol use	Yes	Of those who said they were drinking more than usual, 58.9% were cisgender women.
Specific Populations and Demographic Differences: Demographic Differences by Race/Ethnicity					
1	Hanson^114^	Quantity/frequency	Retrospective report of current drinking in past vs. drinking now	N/A	24.2% of cisgender women reported drinking more now and 50% reported binge drinking since pandemic started; 54.8% had 8+ drinks per week.
2	Hicks^115^	Alcohol use frequency from AUDIT	Pre- and post/during pandemic data	Yes	No differences by sexual identity; cisgender men more likely to decrease alcohol use during pandemic compared to cisgender women. No significant gender differences between cisgender and TNB participants.
Specific Populations and Demographic Differences: Frontline Workers					
1	Beiter^85^	AUDIT	Retrospective recall of pre-pandemic AUDIT	Yes	Cisgender men higher AUDIT than cisgender women; all reported increases in AUDIT compared with pre-pandemic; no gender by time interaction assessed.
2	Hennein^119^	AUDIT-C	N/A	No	Cisgender women were no more likely than men to report AUD symptoms despite higher rates of PTSD.
Specific Populations and Demographic Differences: Couple Relationships, Pregnancy, and Parenting					
1	Ahlers-Schmit^116^	Unclear measurement	Self-perceived changes in alcohol use	N/A	Increases in alcohol use significantly higher postpartum than during pregnancy.
2	McMillan^117^	Epidemic Pandemic Impact Inventory (EPII)^196^	Self-perceived changes in alcohol use	N/A	Almost one-third (28%) reported that they or their partner’s alcohol consumption had increased since the start of the pandemic.
3	Rodriguez^118^	Daily Drinking Questionnaire;^197^ Shortened Inventory of Problems-Alcohol and Drugs scale;^198^ Drinking to cope using two visual analog scales	N/A	Yes	Cisgender men reported significantly more alcohol-related problems than did cisgender women, but drinking levels did not differ by gender. Cisgender women’s drinking was significantly associated with their partner’s drinking and stress; cisgender men’s drinking was unrelated to their partner’s drinking or stress. Cisgender women’s levels of stress were unrelated to their drinking.
Coping, Stress, and Mental Health					
1	Lannoy^94^	AUDIT	Pre- and post/during pandemic data	No	No sex differences, stable AUDIT scores between assessments
2	Tucker^100^	Quantity and frequency; Alcohol problems assessed with the Short Inventory of Problems^195^	Pre- and post/during pandemic data	Yes	Analyses were stratified by gender. Cisgender men’s alcohol use started out higher than cisgender women but declined whereas cisgender women’s stayed static. By time 3, drinking levels were about the same. Both cisgender men and cisgender women had increased alcohol problems over time. Coping and social reasons for drinking and loneliness had distinct associations with alcohol use, alcohol problems, and change over time and these varied by gender.
3	Hennein^119^	AUDIT-C	N/A	No	Cisgender women were no more likely than cisgender men to report AUD symptoms, despite higher rates of PTSD.
4	Cummings^120^	Quantity, frequency, and two items adapted from Drinking Motives Questionnaire^199^	Pre- and post/during pandemic data	Yes	No differences in drinking to cope comparing pre- and during pandemic samples (did not look at gender differences). Significant associations between COVID-19 stress and drinking to cope for cisgender men and women but associations were stronger for men.
5	Devoto^121^	AUDIT-C; Alcohol, Smoking, and Substance Involvement Screening Test^200^	N/A	N/A	Among cisgender women, high-risk alcohol associated with significantly higher levels of depression and anxiety than lower risk use. Cisgender women with moderate drinking risks reported higher levels of social support than cisgender women with high-risk drinking. Almost 17% said that they increased their drug or alcohol use to cope with relationship problems.
6	Graupensperger^122^	Daily Drinking Questionnaire,^197^ binge drinking item from Monitoring the Future questionnaire^201^	N/A	No	No gender differences in rates of binge drinking or number of drinks per week.
7	Helminen^123^	AUDIT-C	N/A	N/A	Nearly half (47.1%) of the sample reported alcohol use consistent with probable AUD.
8	Martinez^124^	Two drinking to cope questions adapted from the Drinking Motives Questionnaire^199^	Pre- and post/during pandemic data	Yes	Among cisgender women, 13.8% reported drinking to cope prior to the pandemic and 15.6% reported drinking to cope during the pandemic, compared to 10.7% before and 17% during the pandemic for cisgender men. These rates were not statistically different. Among cisgender women, those with moderate to severe symptoms of depression or mild symptoms of anxiety were significantly more likely to report drinking to cope. No significant associations were identified for cisgender men.
9	Nesoff^125^	Adapted quantity and frequency items from NSDUH	Self-perceived changes in alcohol use	Yes	Odds of high-risk drinking were significantly elevated for cisgender women when controlling for stress, depressive symptoms, and household job loss. Cisgender men had lower odds of high-risk drinking than cisgender women.
10	Vogel^126^	Short Inventory of Problems—Alcohol and Drugs (SIP-AD)^198^	N/A	No	Sex, race/ethnicity, marital status, and other pandemic-related variables were not associated with SIP-AD scores.
Transgender and Nonbinary Populations					
1	Coakley^101^	Quantity and frequency	Self-report of current drinking at more than one time point	Yes	Pre-pandemic (retrospectively reported), cisgender men drank more than cisgender women who drank more than TNB people. During pandemic, consumption increased across groups, but cisgender men still drank more than cisgender women, who drank more than TNB people. Cisgender men and TNB people had greatest percentage change during pandemic.
2	Hicks^115^	Alcohol use frequency from AUDIT	Pre- and post/during pandemic data	Yes	No differences by sexual identity; cisgender men were more likely than cisgender women to decrease alcohol use during pandemic. No significant gender differences between cisgender and TNB participants.
3	Dyar^127^	Daily drinking questionnaire,^197^ quantity	Self-report of current drinking at more than one time point	No	No significant differences between cisgender women and TNB participants for alcohol use or drinking to cope.
4	Salerno^128^	Indicated if alcohol use had changed since the start of pandemic.	Self-perceived changes in alcohol use	Yes	The effect of increased alcohol use on psychological distress since the start of COVID-19 was nonsignificant for AMAB but was significant for AFAB people.
5	Sumetsky^129^	Quantity and frequency of drinking and number of days of intoxication	Retrospective report of past drinking vs. current drinking	Yes	Compared to cisgender women, cisgender men had more drinks on drinking days during pandemic, and more days intoxicated pre-pandemic. There were no significant differences for TNB people.
6	Zhang^130^	AUDIT	Self-perceived changes in alcohol use	Yes	Transgender and GNC people had lower problem drinking, and were less likely to have perceived increase in their drinking during COVID-19 than cisgender participants.
7	Akré^131^	PROMIS Alcohol Use Negative Consequences 7-item short-form scale	Self-report of changes in alcohol consumption due to the pandemic	Yes	No substantial difference in rates of increased alcohol use between transgender and cisgender, straight respondents, but some elevated use among cisgender, sexual minority respondents.
Sexual Minority Women					
1	Dyar^127^	Daily drinking questionnaire,^197^ quantity	Self-report of current drinking at more than one time point	No	No significant differences between cisgender women and TNB participants for alcohol use or drinking to cope.
2	Salerno^128^	Indicated if alcohol use had changed since the start of pandemic.	Self-perceived changes in alcohol use	Yes	The effect of increased alcohol use on psychological distress since the start of COVID-19 was non-significant for AMAB but significant for AFAB people.
3	Dyar^132^	AUDIT, Drinking motives, Brief Young Adult Alcohol Consequences Questionnaire^202^	Self-perceived changes in alcohol use	N/A	Nearly all participants reported more anxiety and depression in the past month compared to before the pandemic; approximately half also reported increases in alcohol and cannabis use.
4	Peterson^133^	AUDIT	Self-perceived changes in alcohol use	Yes	SMW more likely to report alcohol use increased since beginning of pandemic than SMM and cisgender heterosexual women

Within each section, studies are listed in the order in which they are cited. Some studies are listed in more than one section.

*Note:* AFAB, assigned female at birth; AMAB, assigned male at birth; AUD, alcohol use disorder; AUDIT, Alcohol Use Disorders Identification Test; AUDIT-C, Alcohol Use Disorders Identification Test-Consumption; BRFSS, Behavioral Risk Factor Surveillance System; COVID-19, coronavirus disease 2019; *DSM-V, Diagnostic and Statistical Manual of Mental Disorders*, fifth edition; GNC, gender nonconforming; N/A, not applicable (in the Gender Differences column, N/A indicates that the sample includes cisgender women only); NSDUH, National Survey on Drug Use and Health; PROMIS, Patient-Reported Outcomes Measurement Information System; PTSD, post-traumatic stress disorder; SBIRT, screening, brief intervention, and referral to treatment; SIP-AD, Short Inventory of Problems–Alcohol and Drugs; SMM, sexual minority men; SMW, sexual minority women; TNB, transgender and nonbinary.
